# Vitamin D supplementation in the prevention and management of major chronic diseases not related to mineral homeostasis in adults: research for evidence and a scientific statement from the European society for clinical and economic aspects of osteoporosis and osteoarthritis (ESCEO)

**DOI:** 10.1007/s12020-017-1290-9

**Published:** 2017-04-07

**Authors:** Luisella Cianferotti, Francesco Bertoldo, Heike A. Bischoff-Ferrari, Olivier Bruyere, Cyrus Cooper, Maurizio Cutolo, John A. Kanis, Jean-Marc Kaufman, Jean-Yves Reginster, Rene Rizzoli, Maria Luisa Brandi

**Affiliations:** 1 Bone Metabolic Diseases Unit, Department of Surgery and Translational Medicine, University Hospital of Florence and University of Florence, Florence, Italy; 2 Department of Medicine, University of Verona, Verona, Italy; 3 Department of Geriatrics and Aging Research, University Hospital Zurich and University of Zurich, Zurich, Switzerland; 4 Epidemiology and Public Health, University of Liege, CHU Sart Tilman, Liege, 4000 Belgium; 5 MRC Lifecourse Epidemiology Unit, University of Southampton, Southampton, Hants UK; 6 Research Laboratory and Academic Division of Clinical Rheumatology, Department of Internal Medicine, University of Genova, Genoa, Italy; 7 Centre for Metabolic Bone Diseases, University of Sheffield Medical School, Sheffield, UK; 8 Institute for Health and Aging, Catholic University of Australia, Melbourne, VIC Australia; 9 Department of Endocrinology and Unit for Osteoporosis and Metabolic Bone Diseases, Ghent University Hospital, Ghent, Belgium; 10 Department of Public Health, Epidemiology and Health Economics, University of Liège, CHU Sart-Tilman, Liège, Belgium; 11 Service of Bone Diseases, Geneva University Hospitals and Faculty of Medicine, Geneva, Switzerland

**Keywords:** Cholecalciferol, Cancer, Diabetes, Mortality, Cardiovascular diseases, Autoimmune diseases

## Abstract

**Introduction:**

Optimal vitamin D status promotes skeletal health and is recommended with specific treatment in individuals at high risk for fragility fractures. A growing body of literature has provided indirect and some direct evidence for possible extraskeletal vitamin D-related effects.

**Purpose and Methods:**

Members of the European Society for Clinical and Economic Aspects of Osteoporosis and Osteoarthritis have reviewed the main evidence for possible proven benefits of vitamin D supplementation in adults at risk of or with overt chronic extra-skeletal diseases, providing recommendations and guidelines for future studies in this field.

**Results and conclusions:**

Robust mechanistic evidence is available from in vitro studies and in vivo animal studies, usually employing cholecalciferol, calcidiol or calcitriol in pharmacologic rather than physiologic doses. Although many cross-sectional and prospective association studies in humans have shown that low 25-hydroxyvitamin D levels (i.e., <50 nmol/L) are consistently associated with chronic diseases, further strengthened by a dose-response relationship, several meta-analyses of clinical trials have shown contradictory results. Overall, large randomized controlled trials with sufficient doses of vitamin D are missing, and available small to moderate-size trials often included people with baseline levels of serum 25-hydroxyvitamin D levels >50 nmol/L, did not simultaneously assess multiple outcomes, and did not report overall safety (e.g., falls). Thus, no recommendations can be made to date for the use of vitamin D supplementation in general, parental compounds, or non-hypercalcemic vitamin D analogs in the prevention and treatment of extra-skeletal chronic diseases. Moreover, attainment of serum 25-hydroxyvitamin D levels well above the threshold desired for bone health cannot be recommended based on current evidence, since safety has yet to be confirmed. Finally, the promising findings from mechanistic studies, large cohort studies, and small clinical trials obtained for autoimmune diseases (including type 1 diabetes, multiple sclerosis, and systemic lupus erythematosus), cardiovascular disorders, and overall reduction in mortality require further confirmation.

## Introduction

Adequate vitamin D status is undoubtedly necessary for the maintenance of optimal mineral and skeletal homeostasis, as well as for the prevention and cure of secondary hyperparathyroidism, rickets and osteomalacia [1]. The measurement of serum levels of 25-hydroxyvitamin D [25(OH)D] is used both to determine vitamin D status and to estimate the benefit of vitamin D supplementation [2]. According to different guidelines, the thresholds for serum 25(OH)D have been set at 50 or 75 nmol/l (i.e., 20 or 30 ng/ml) for bone health [3–6]. Levels of 25(OH)D beyond these thresholds do not appear to confer additional benefits for mineral homeostasis [1, 7]. According to international recommendations, vitamin D status has to be determined in subjects at risk for disorders of bone and mineral metabolism [3, 5]. Nonetheless, the wide availability of 25(OH)D commercial assays has caused the requests for the assessment of vitamin D status to increase markedly in recent years and, according to the above-described thresholds, many subjects have been defined as vitamin D deficient [8, 9]. Alternatively, current recommendations of the International Osteoporosis Foundation with 600 IU per day in younger and middle aged adults and 800 IU per day in older adults ensure that over 97% of individual reach a replete vitamin D status with 20(OH)D levels of 20 ng/ml [2, 10, 11]. Nonetheless, while this mainly applies to the North American individuals, it might not apply to populations that do not usually fortify their foods with vitamin D thus displaying lower vitamin D levels [2, 11].

Several reports have shown that vitamin D deficiency is associated with an array of chronic diseases [12, 13]. Yet, the causal effect of low serum levels of 25(OH)D on the onset and progression of diseases that are unrelated to mineral homeostasis, has yet to be demonstrated in large clinical trials. Most evidence is still based on observational studies [association with ultraviolet B radiation (UVB) exposure, 25(OH)D levels] [12, 13].

Large, randomized controlled clinical trials assessing the benefits of sufficient dose of vitamin D supplementation on different chronic diseases outside the skeleton as primary endpoints are still lacking, and no specific thresholds have been defined in this field for each different effect. Notably, meta-analyses have limitations because of the selection of studies, quality of endpoint assessment, analytical aspects and interpretation of the results [14, 15]. Moreover, there is still much uncertainty whether achieving values of serum 25(OH)D greater than the recommended thresholds may lead to any benefit in overall health [7, 16]. Nonetheless, assessment of vitamin D status and vitamin D supplementation are nowadays widely prescribed by different specialists and general practitioners for a variety of chronic conditions not classically linked to mineral and bone metabolism abnormalities [17].

Members of the European Society for Clinical and Economic Aspects of Osteoporosis and Osteoarthritis, along with experts in the field of vitamin D, convened a meeting in February 2016 to broadly review the main evidence for possible proven benefits of vitamin D supplementation in adults at risk of or with overt chronic extra-skeletal diseases, providing recommendations and guidelines for future studies in this field.

Therefore, the aims of this paper were: to summarize and highlight the main available evidence of vitamin D-related extraskeletal benefits, reviewed in detail elsewhere, mainly ensuing from systematic reviews of large cohort data, small randomized controlled trials (RCTs), and meta-analyses of clinical trials; to give recommendations for clinical practice; to issue the research agenda on the possible advantages of vitamin D treatment on extra skeletal chronic diseases, focusing on cardiovascular diseases and overall mortality, diabetes mellitus, main autoimmune diseases, and cancer.

## Molecular rationale (mechanistic data) for possible extraskeletal vitamin D-mediated effects

**Fig. 1:**
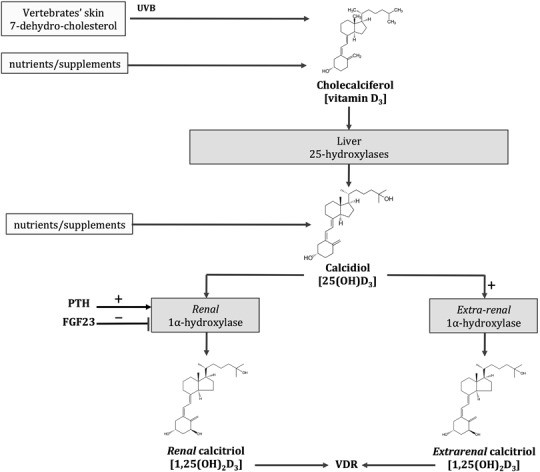
Vitamin D metabolism. Endogenous or exogenous cholecalciferol and calcidiol are the inactive precursors of the biological active hormone calcitriol. Calcitriol, classically produced in the kidneys under the positive and negative regulation of parathyroid hormone (PTH) and fibroblast growth factor 23 (FGF23), respectively, can also be synthesized in extra-renal tissues, where its production is mainly driven by the substrate, 25 hydroxyvitamin D (25(OH)D). The nearly ubiquitously expressed vitamin D receptor (VDR) mediates calcitriol actions in skeletal and extra-skeletal tissues

The biologically active form of vitamin D, calcitriol [1,25(OH)_2_D], is a multifunctional steroid hormone produced by the kidney (Fig. 1). It exerts its actions through the activation of the vitamin D receptor (VDR), a nuclear receptor almost ubiquitously expressed in most vertebrate cells, but mostly present in the intestine, where it stimulates active calcium absorption [5]. As demonstrated by in vitro and in vivo evidence, calcitriol can also be synthesized in a series of tissues in normal or pathologic conditions. Extra-renal calcitriol mainly acts in an autocrine or paracrine manner, in order to modulate functions not classically related to mineral homeostasis. Whilst renal calcitriol production is regulated by parathyroid hormone (PTH) and fibroblast growth factor 23, two hormones that respectively enhance or inhibit its production, the synthesis of extra-renal calcitriol is driven by the bioavailable substrate, i.e., serum 25(OH)D. Concentrations of free or bioavailable 25(OH)D in the local circulation at the target tissues are also regulated by the levels of vitamin D binding protein (VDBP) [18]. The ubiquitous presence of the VDR and the possible production of extra-renal calcitriol driven by the concentration of the non-active pre-hormone, 25(OH)D, constitute the physiological conditions for the potential extra-skeletal effects of calcitriol and suggest a possible role for parental vitamin D or 25(OH)D in maintaining or enhancing these processes [19].

A recent RCT conducted in a small group of healthy adults has demonstrated that any increase in vitamin D levels would significantly affect the expression of genes belonging to several pathways involved in the pathogenesis of major chronic diseases [20].

Many in vitro studies have shown the effects of the active hormone calcitriol on cells belonging to extra-musculoskeletal tissues expressing both the VDR and 1alpha hydroxylase (1αOHase), the enzyme which ultimately activates the pro-hormone 25(OH)D [21]. The model of the VDR knockout mouse, which develops hyperparathyroidism and rickets soon after weaning, has reproduced in vivo the ligand dependent and independent VDR-mediated effects also on organs not related to mineral homeostasis, such as skin, cardiovascular/renin-angiotensin system, and metabolic system [22, 23]. The global VDR knockout mice also develop alopecia, hypertension, impaired insulin secretion, skeletal muscle fiber atrophy with motor deficits, left ventricular hypertrophy and failure, and cardiac fibrosis [22–28]. These mice are more prone to skin cancer formation and impaired response to injury [29]. Many in vitro studies have shown the direct effects of calcitriol in modulating the functions of cells belonging to different human and animal tissues.

## Vitamin D, cardiovascular diseases, and mortality: evidence

The observations that both systolic and diastolic blood pressure (SBP and DBP, respectively) increase with the distance from the equator [30], and that seasonality for major events such as hospitalizations and in-hospital death and mortality in a large dataset [31], suggested that vitamin D could play a role in modulating cardiovascular health and mortality.

At a mechanistic level, several lines of evidence link vitamin D to cardiovascular health. First, the VDR has been found to be present in key tissues linked to cardiovascular health, such as myocardial, endothelial, smooth muscle, and pancreatic beta cells, as well as macrophages [32, 33]. Second, in situ production of the active hormone by the 1αOHase has been confirmed in all of the same tissues, suggesting a requirement for calcitriol [32, 33]. Third, the VDR knock-out mouse has been shown to suffer from hypertension and congestive heart failure [22], further supported by the VDR mediated downregulation of the expression of renin, which is one of the major determinants in cardiovascular risk [34]. Forth, deletion of the VDR in cardiomyocytes resulted in ventricular hypertrophy among mice [25]. Finally, in humans, several large cohort studies have demonstrated that low serum 25(OH)D levels are predictive of an increased risk of incident hypertension [35, 36], myocardial infraction [37], and sudden cardiovascular death [38, 39]. Notably, regarding a desirable 25(OH)D range for optimal cardiovascular health, these large cohort studies suggested that for incident hypertension in both men and women, cardiovascular mortality and all-cause mortality, individual who had 25(OH)D levels between 50 and 130 nmol/l may have the lowest prospective risk [2]. In a prospective cohort study of 3258 consecutive patients of both genders and a mean age of 62 years scheduled for coronary angiography, both all-cause and cardiovascular mortality increased in a dose-dependent manner with decreasing quartiles of baseline serum 25(OH)D levels [38]. In a prospective study of elderly men, low serum 25(OH)D was associated with a substantial excess risk of death compared to 25(OH)D values greater than 50–70 nmol/l [40]. Consistent findings come from the Longitudinal Study Amsterdam where among 1317 senior men and women (age: 65–85 years) those with deficient serum 25(OH)D levels had a significantly higher risk of overall mortality (HR 1.46; 95% CI 1.12–1.91 for 25(OH)D <25 nmol/l and HR 1.24; 95% CI 1.01–1.53 for 25(OH)D 25–49.9 nmol/l) [41].

Extending to peripheral artery disease, an inverse dose-response relationship was observed cross-sectionally between 25(OH)D status and peripheral arterial diseases among individuals age 40 years and older in the large US population-based NHANES III (2001–2004) study [42]. While a Mendelian randomization study has pointed to a possible causal relationship between 25(OH)D levels and hypertension by meta-analyzing data for up to 108,173 individuals from 35 studies [43], a similar study has failed to confirm a causal relationship between serum 25(OH)D levels and mortality rates [44].

Several RCTs, albeit small and short-term, have assessed the effects of vitamin D supplementation on cardiovascular parameters among vitamin D deficient individuals. In a group including 18 subjects with hypertension, randomized to be exposed to UVB or ultraviolet A radiation thrice weekly over a period 6 weeks at suberythematous doses, both systolic blood pressure (SBP) and diastolic blood pressure (DBP) decreased by 6 mmHg in the UVB-treated group [45]. Furthermore, a subsequent study, carried out on a group of 148 community-dwelling elderly women, demonstrated that the administration of 800 IU of cholecalciferol (plus calcium) led, in the short term (8 weeks), to a mean significant decrease of 13 and 6 mmHg in SBP and DBP, respectively, being more effective than calcium alone [46]. In a pharmacokinetic study comparing calcidiol and cholecalciferol, 20 healthy postmenopausal women with low vitamin D status (mean age 61.5 years) were randomized to receive 20 mcg of calcidiol or 20 mcg (i.e. 800 IU) of cholecalciferol, leading to a period of 4 months to mean serum 25(OH)D targets of 174 and 76 nmol/l, respectively. In the group of women receiving calcidiol, blood pressure was significantly lower at each measured time-point after the 1st week of treatment, with sustained mean 5.7 mmHg decrease in SBP over 4 months of treatment demonstrated in the group of women receiving calcidiol vs. no change in the ones receiving cholecalciferol (*P* = 0.002), independently of age, body mass index (BMI), and baseline SBP [47].

In a short-term 8-week trial among 200 individuals with hypertension and serum 25(OH)D levels less than 75 nmol/l (mean 52.9 nmol/l) were randomized to receive 2800 IU of cholecalciferol or placebo for 8 weeks [48] and vitamin D treatment did not decrease blood pressure [48]. The authors hypothesized that their null finding may have been due to the fact that too many participants were not vitamin D deficient at baseline. This is consistent with their post-hoc subgroup analysis among participants who were vitamin D deficient at baseline, who did have a significant benefit on the renin-angiotensin system with vitamin D treatment group based on a reduction in their plasma aldosterone concentration [49].

At the level of published meta-analyses of clinical trials on the effect of vitamin D supplementation and blood pressure, where blood pressure was measured as primary or secondary end-point, or simply measured, a benefit of vitamin D supplementation on blood pressure could not be demonstrated [50–54]. The most recent meta-analysis included 46 trials (total of 4541 participants) and suggested a null effect of vitamin D on blood pressure, irrespective of subgroup [54].

At the level of published meta-analyses of clinical trials on the effect of vitamin D supplementation and mortality, the most recent Cochrane meta-analysis focused on all-cause mortality and cancer mortality among 75,927 individuals from 38 studies on all-causes mortality and 44,492 individuals from 4 studies on cancer-mortality. This analysis showed a significant 6% reduction in all cause mortality and a 12% reduction in cancer mortality in supplemented subjects if compared with placebo or calcium [55]. In a sequential meta-analysis taking into account RCTs with vitamin D supplementation of any duration and quality, the authors found a significant 4% reduction in all-cause mortality [56]. In order to assess the effect on single causes of mortality, another meta analysis including the randomized evaluation of calcium or vitamin D (RECORD) trial, and additional 21 RCTs among seniors, concluded that vitamin D supplementation might protect against cardiac failure in older individuals, but does not appear to protect from stroke or myocardial infarction [57].

Desirable 25(OH)D levels for optimal risk reduction in mortality have been explored in several epidemiologic studies [38, 58–63], most of which suggested a continuous inverse relationship between increasing values of 25(OH)D and a lower risk of mortality. In some studies and reviews, however, a U-shape or reverse J-shaped relationship has been described with an increased risk of mortality both at low and higher levels of 25(OH)D [60, 64–67].

In summary, evidence that link vitamin D to cardiovascular health is limited to mechanistic studies, large cohort studies and small clinical trials among vitamin D deficient adults. Large clinical trials with a sufficient dose of vitamin D, ideally tested among individuals at risk of vitamin D deficiency, are missing for blood pressure, any major cardiovascular events and mortality. Two ongoing trials are addressing this gap with available results in 2018 [VITAL Study [68], DO-HEALTH trial [69]. Both trials test 2000 IU vitamin D against placebo with VITAL addressing major cardiovascular events and DO-HEALTH blood pressure as primary endpoints. Based on available data, effects are most likely expected in deficient individuals and both trials have undertaken recruitment strategies to target adults (VITAL: age 50+; DO-HEALTH: age 70+) at risk of vitamin D deficiency, although they did not select for vitamin D deficient subjects (i.e., serum 25(OH)D less than 20 ng/ml). All ongoing large-scale multicenter clinical trials with predefined cardiovascular endpoints are listed in Table 1.

**Table 1:** Ongoing large-scale randomized controlled trials in subjects aged 50 years or more, to assess vitamin D-mediated effects on multiple health outcomes

Name	Place	Participants	Dose	Main outcomes	Current state	Results expected
VITAL	U.S.	20,000 men: 50+ women: 55+	2000 IU D_3_ daily	Cancer, cardiovascular disease	Recruitment to finish end of 2012	2017
FIND	Finland	18,000 men: 60+ women: 65+	1600 IU D_3_ daily		Recruitment started in spring, supplementation to start in autumn	2020
ViDA	New Zealand	5100, 50+	100,000 IU D_3_ a month (200,000 IU in June)		Recruitment to finish in 2016	2017
DO-Health	Five European countries	2150, 70+	2000 IU D_3_ daily or 3600 IU daily	Infections, fractures, blood pressure, cognitive function, lower extremity function	Recruitment finished in 2014	2018
VIDAL	UK	20,000, 65–84	60,000 IU D_3_ monthly	Longevity and others	Planned 2-year feasibility study on 1600 patients is recruiting	2020 (If main study gets go-ahead)

## Vitamin D, type II diabetes and obesity: evidence

Many studies have shown an association of type II diabetes (T2D), metabolic syndrome and obesity with a poor vitamin D status [70]. After the first observation that vitamin D status itself affects pancreatic secretion of insulin after proper stimulus in rats [71], further experimental studies have demonstrated that pancreatic beta-cells express the VDR and 1αOHase [70] and that calcitriol directly stimulates insulin production by pancreatic islets [72], modulates peripheral insulin sensitivity and systemic inflammation in vitro and in vivo in animal models [70]. In humans, a polymorphism of the VDR possibly impairing the response to calcitriol has been shown to be a significant and positive predictor of T2D and myocardial infarction [73].

In the NHANES III, serum 25(OH)D levels were inversely correlated with the prevalence of T2D and measures of insulin resistance in a dose-dependent pattern in some, but not in all, ethnic groups (i.e., non-Hispanic whites and Mexican–Americans), without correlating with beta-cell function [74]. In the large longitudinal study of the Nurses Health Study, after adjustment for all the possible co-variates, the risk of developing T2D was reduced by 33% in women with higher intake of vitamin D and calcium (>1200 mg and >800 IU daily, respectively) [75]. As far as the complications of diabetes are concerned, serum 25(OH)D levels were shown to be an independent predictor of macrovascular and microvascular problems in patients with overt T2D [76].

A meta-analysis of longitudinal observation studies by Song et al. included 21 studies with 76,000 participants and calculated the risk of developing T2D according to baseline vitamin D status [77]. The risk of developing T2D was reduced by 38% in the subjects in the highest tertile for serum 25(OH)D levels as compared with those in the lowest tertile, with little heterogeneity among studies. The association was consistent regardless of various baseline variables, such as diagnostic criteria for diabetes, duration of follow-up, or study size, and remained significant after adjustment for BMI and intermediate biomarkers. A linear trend analysis showed that a 4 ng/ml increment in 25(OH)D levels corresponded to a 4% lower risk of developing T2D [77]. Nonetheless, two Mendelian randomization studies have failed to demonstrate a causal relationship between a low vitamin D status and T2D or obesity, respectively [78, 79]. Moreover, a systematic review and meta-analysis has demonstrated that vitamin D and calcium supplements had no effects on adiposity in adults [80]. The authors concluded that the effort to increase 25(OH)D levels by means of supplementation might not be beneficial to reduce the risk of T2D or obesity [78–80].

The evidence from intervention trials assessing the influence of vitamin D supplementation in T2D is still scarce and mostly comprises post-hoc analyses. These RCTs were mainly designed for non-glycemic outcomes, they were often too short and the dose of administered vitamin D was heterogeneous, as reported in a recent systematic review [81]. Whilst it appears that vitamin D supplementation has a neutral effect on glycemic outcomes in individuals with normal glucose tolerance and in people with established T2D at baseline, its potential effect seems to be more prominent in those people who are at increased risk for diabetes [70].

RCTs specifically designed to assess the effect of vitamin D supplementation on T2D risk and insulin sensitivity (homeostatic model assessment of insulin resistance, i.e., HOMA) are still a few. In one of these studies performed in healthy adults at increased risk for T2D with low vitamin D status (≤55 nmol/l), only the subgroup of subjects with prediabetes had an advantage from daily cholecalciferol, administered at a dosage sufficient to target serum levels of 25(OH)D of >75 nmol/L in terms of increase in insulin sensitivity [82].

In healthy adult individuals with low 25(OH)D levels, supplementation with high dose vitamin D2 (50,000 IU/week) had no effect increasing insulin secretion and insulin sensitivity in the short-term (12 weeks) [83]. With respect to at-risk subjects, in a recently published long-term RCT involving 511 subjects (mean age 62 years) with prediabetes within the Tromso cohort carried out in the years 2008–2015, 20,000 IU/week of cholecalciferol did not prevent the progression to overt T2D [84].

Because of the potential adverse effects of high dose vitamin D, one ongoing placebo-controlled study (ie. D2d study) will test both the long-term safety and efficacy of daily high-dose vitamin D supplementation (4000 IU/day) on lowering the risk of progression to overt diabetes in people with increased risk for this chronic disease [85].

In conclusion, no evidence exists, so far, that administering vitamin D could reduce T2D or obesity in the general population. The results obtained in subjects with prediabetes require further confirmation by larger and longer RCTs. Since higher doses are employed, studies on safety are also needed.

## Vitamin D and autoimmune diseases: evidence

Calcitriol is a regulator of the immune system [86, 87]. Whilst it exerts stimulatory effects on innate immunity, which is aspecific and implicated in the defense against infections, it also modulates the effectors of adaptive immunity, which is acquired and antigen-specific [88]. The observations that the geographic prevalence of autoimmune diseases such as multiple sclerosis (MS), type 1 diabetes mellitus (T1D), rheumatoid arthritis (RA) and other rheumatic diseases increases with the distance from the equator or changes with seasonality, as well as the worsening of these diseases in conditions of low ultraviolet radiation (UV) exposure, have raised the hypothesis that vitamin D could play a role in the pathogenesis of these diseases [89–91]. Indeed, the transcriptomic profile of the immune system in man varies with season and is shifted towards a pro-inflammatory state in wintertime [92]. Nonetheless, the observation that UV irradiation can repress the development of experimental autoimmune encephalomyelitis (EAE), a murine model of MS, independently of 25(OH)D levels, has in part downsized the belief that UV could act through vitamin D production to determine the above-listed effects [93].

Calcitriol has been shown to modulate in vitro the activity of key players of the immune system, such as antigen presenting cells and T-lymphocytes [94]. Calcitriol inhibits the type T1 helper cell function by suppressing inflammatory cytokine production (IFN-γ and IL-2), IL-17 producing T-cells, and dendritic cell differentiation, whilst it enhances the production of cytokines by the type T2 cells (Th2) such as IL-10 and the activity of regulatory T (Treg) cells [94]. In addition, calcitriol downregulates aromatase expression and inflammatory cytokines in human macrophages [95]. These effects lead to an important defensive mechanism against inflammation and improvement of tolerogenic phenotype. These findings, together with the fact that individuals with autoimmune diseases often display a poor vitamin D status as compared with controls, have led to hypothesize a potential immunomodulatory effect for vitamin D and to study the immune system in the VDR knockout mouse model. Indeed, mice devoid of VDR failed to demonstrate gross immune abnormalities, except for impaired macrophage chemotaxis and a lower response to anti-CD3 stimulation [26]. Moreover, vitamin D receptor knockout mice were unexpectedly protected from low-dose streptozotocin–induced diabetes mellitus and EAE was less severe in VDR null mice [26, 96]. These immune defects were rescued by means of a diet rich in calcium, lactose and phosphate, demonstrating that they were an indirect effect of VDR disruption and that, although calcitriol is a possible pharmacologic or physiologic immunomodulator, these actions are redundant in vivo [26]. Conversely, in animal models of autoimmune disease, a benefit of the administration of vitamin D, calcitriol or calcitriol analogs on preventing the onset or blunting the disease progression via direct modulation of immune cells (i.e., induction of tolerogenic dendritic cells) has been demonstrated [86, 97].

Epidemiological studies have shown an association between serum 25(OH)D levels and the prevalence, incidence, severity and progression of many autoimmune diseases [88]. Indeed, higher levels of 25(OH)D have been associated with a decrease in the likelihood of developing autoimmune diseases such as MS, RA, T1D [98], especially when taken early in life [86]. A systematic review and meta-analysis has demonstrated that, despite heterogeneity, poor vitamin D status was associated to an increased risk of developing MS [99]. Moreover, two Mendelian randomization studies have recently pointed to a likely causal relationship between poor vitamin D status and the risk of MS [100, 101]. In MS, low vitamin D status has been shown to be an independent early predictor of disease activity and progression [102], in particular in patients being treated with IFN beta-1b [103]. In addition, IFN beta was indeed more effective in MS in the presence of high levels of 25(OH)D [104].

The potential role of calcitriol as immunomodulator/immune-suppressor gave rise to the hypothesis that calcitriol or other active vitamin D analogs, such as alfa-calcidiol, might be used as a pharmacologic agent to prevent autoimmune disease in high-risk individuals, or to treat overt autoimmune diseases and to protect transplanted organs from rejections. However, calcitriol must be administered in high doses to elicit an immunomodulatory effect and suppress proinflammatory cytokines. For this reason, it cannot be used in humans. Non-hypercalcemic analogs of calcitriol are currently under investigation.

The fact that calcitriol can be synthesized by immune cells because of the expression of 1αOHase, which is regulated by its substrate, i.e., 25(OH)D, has been exploited to support the concept that vitamin D supplementation with parental vitamin D compounds could be considered for the pharmacologic adjuvant treatment of autoimmune diseases [21].

A few RCTs have demonstrated that supplementation with the pro-hormone vitamin D has similar effects with respect to calcitriol on cell-mediated immunity. Indeed, monthly supplementation with high dose (140,000 IU) vitamin D3 increased significantly peripheral regulatory T-cells in adult healthy donors in the short term of 3 months, as compared to placebo [105]. Similarly, daily high dose vitamin D3 (4000 IU daily) led to a significant decrease in CD4 cytotoxic T-cell activation compared to low dose vitamin D3 (400 IU/day) [106].

In patients relapsing-remitting MS (RRMS, study group 94 subjects) high dose vitamin D intake (50,000 IU every 5 days for 3 months) along with IFN-β treatment led to a significant increase in mental quality of life vs. placebo [107]. An even higher dose of cholecalciferol (10,400 IU/day) was proven to be safe and well tolerated, at least in the short-term (6 months), and led to pleiotropic immunomodulatory effects (decreased production of IL-17 and proportion of effector memory CD4+ cells), with a concomitant increase in central memory CD4+ cells [108]. These latter findings confirm previous results obtained in similar randomized controlled studies in MS (as reviewed in 110). However, larger and long-term studies are necessary to confirm the efficacy and safety of vitamin D supplementation in MS [109].

Vitamin D supplementation early in life (2000 IU daily) has been shown to reduce the risk of developing T1D in at-risk subjects in a retrospective study in northern Finland, where individuals are likely to be vitamin D deficient for most part of the year [110]. A recent study in mice indicated that high dose parental vitamin D3 reduced the incidence of diabetes in a mouse model, which spontaneously develops diabetes (non-obese diabetic, i.e., NOD mouse), when the vitamin was administered at high doses and lifelong from 3 weeks of age [111]. Randomized controlled longitudinal studies are ongoing to assess this effect of vitamin D in at-risk human young populations. As far as the early stages of disease are concerned, both alfacalcidiol and parental vitamin D3 (70 IU/Kg body weight/day) have been proven to be effective on residual beta-cell function in latent autoimmune diabetes in adults and improved suppressor function of regulatory T cells in patients with T1D, respectively, in recently published RCTs [112, 113]. Data on larger groups of individuals confirming these results are still missing in T1D.

The demonstration of circannual rhythms in RA and systemic lupus erythematous (SLE) and the lower risk of developing RA in the case of higher UVB exposure, suggest that a possible association with vitamin D status might exist [114–116]. However, a post-hoc analysis of the Women Health Initiative study failed to show an association between RA and solar irradiation, and suggested an increased incidence of RA with higher vitamin D exposure of just 440 IU/day vs. placebo [117]. Contradictory results arose from meta-analyses assessing the association between vitamin D intake and risk of RA and SLE in women [118, 119], whereas a recent meta-analysis, including 24 cross-sectional studies and involving 3489 subjects, showed a negative association between 25(OH)D levels and disease activity in subjects with RA [120]. Thus far, no RCTs have been carried out to definitively demonstrate a causal relationship between RA and vitamin D status by assessing the effects of vitamin D supplementation on the course of the disease. In SLE, a RCTs comparing the effect of daily supplementation with 2000 IU cholecalciferol against placebo in patients with active disease demonstrated that daily supplementation with cholecalciferol administered over a period of 1 year led to a significant improvement in disease activity, along with a significant decrease in inflammatory markers [121]. Unfortunately, these results have not been confirmed in a crossover trial with a 2-year duration, in which 32 women with SLE were randomized to different regimens of cholecalciferol (25,000 IU monthly or 300,000 IU initial bolus followed by 50,000 IU monthly). The higher dose was not effective in modulating disease activity, despite an increase in the number of Treg cells [122, 123].

A recent Cochrane meta-analysis has shown that there is insufficient evidence to consider vitamin D as a possible relief for several conditions characterized by chronic pain [124].

Although the effects of calcitriol on the modulation of the immune system in vitro are consistent, it remains to be clarified whether these effects have been observed because of the higher pharmacologic doses administered in culture and whether they can be reproduced in vivo. Many of these effects seem to arise directly from the VDR-mediated actions of calcitriol, as demonstrated in animal models. While the in vivo administration of active vitamin D at high doses is not possible because of the hypercalcemic effects, it is not clear to what extent a supplementation of parental vitamin D compounds (cholecalciferol and calcidiol) or non-hypercalcemic calcitriol analogs, could lead to a modulation of the immune system, taking advantage of the possible induction of 1αOHase present in the immune cells.

Promising results have recently been obtained in individuals with MS and subjects at high risk for T1D with vitamin D deficiency by means of larger doses of parental compounds (D3) or active vitamin D analogs. However, larger long-term RCTs assessing safety along with efficacy are needed. The evidence for potential benefits in rheumatic autoimmune diseases is still lacking and requires RCTs possibly carried out during the early stages of the disease to control progression, and in later stages for the prevention of flare-ups.

## Vitamin D and cancer: evidence

Calcitriol controls cellular proliferation and differentiation in vitro. Calcitriol induces apoptosis, autophagy and growth arrest of cancer cells or their progenitors, enhances DNA repair and antioxidant protection, and modulates the immune system to react against cancer [125]. Thus, active vitamin D may inhibit cancer progression and metastasis [125]. These effects are mediated by the VDR, which is expressed by tumor cells along with 1αOHase. This, in turn, is responsible for the local conversion of the pro-hormone 25(OH)D into the biologically active vitamin D. Unfortunately, the capacity to hydroxylate the direct precursor of active vitamin D is progressively lost by cancer cells, especially in prostate cancer [125]. Mice devoid of the VDR were more prone to skin cancer in response to chemical carcinogens or UVB irradiation, although they did not spontaneously develop tumors [126]. In a murine model of bone metastasis, vitamin D deficiency favoured the growth of injected prostate cancer cells in bone likely changing the bone microenvironment [127]. In this regard, it has been also argued that calcitriol could play a role in modulation of osteoblasts, osteoclasts and quiescent cancer cells within the pre-metastatic niche in bone and possibly prevent bone metastases [128].

In humans, epidemiological data have shown an increased prevalence of several types of cancer in the northern areas of the northern hemisphere, suggesting an inverse trend with the amount of UV exposure [129]. Many studies have shown an increased prevalence of vitamin D deficiency in individuals with cancer vs. controls and an association between low vitamin D status and increased risk of developing various tumors, such as breast, prostate, and colon cancer, as well as disease severity [129–132]. Thus, it was suggested that higher serum levels of 25(OH)D and, for prostate cancer, higher serum levels of 1,25(OH)_2_D, would inhibit colorectal, breast and prostate carcinogenesis [130–133]. In particular, levels of 25(OH)D far above the thresholds generally advised for the maintenance of bone and mineral homeostasis (i.e., >50 ng/ml) would prevent cancer. Based on these observational data, it was estimated that even modest increase in serum 25(OH)D levels to 40–60 ng/ml would have prevented 58,000 new cases of breast cancer and 49,000 new cases of colon cancer in the United States and Canada each year, with correspondent reduction in cancer-related mortality rates [134]. Surprisingly, a recent pooled analysis demonstrated an increased risk of prostate cancer along with higher vitamin D intake [135], further confirming that no definitive conclusions can be drawn by observational studies in this field.

Meta-analyses have assessed the association between VDR polymorphisms and cancers, showing that variants of the VDR or higher levels of VDBP were associated with an increased risk for certain types of cancer [136, 137]. These results suggest that, besides 25(OH)D levels, the VDR-mediated response to active vitamin D or the VDBP-regulated exposure to active vitamin D could also be associated with the risk and progression of cancer, and could be considered additional variables in determining the vitamin D-related cancer risk and progression of cancer.

Early studies examined the risk of cancer by means of secondary analyses of previous RCTs, including the Women Health Initiative, and reported no significant cancer risk reduction in individuals supplemented with vitamin D [138].

Some RCTs have been carried out to specifically assess whether vitamin D supplementation can indeed prevent cancer. A group of 1179 community-dwelling women was randomized to receive 1400–1500 mg supplemental calcium plus 1100 IU/day vitamin D3, calcium alone or placebo, and followed-up for a 4-year period. In the intention-to-treat analysis, the supplementation of vitamin D and calcium was shown to be effective in reducing all-cancer risk, with a cancer-free survival 77% higher in the calcium-vitamin D group vs. placebo [139].

A meta-analysis took into account RCTs, prospective cohort studies and nested case–control studies mainly performed in older women, with data on risk of cancer and cancer-related mortality (three studies), or fracture outcomes (16 studies) [140], It was shown that, whilst combined calcium and vitamin D supplementation (1000 IU/day) may reduce the risk for all cancers, with a dose–response relationship observed for colon cancer but not for breast and prostate cancer, surprisingly, higher concentrations of serum 25(OH)D were associated with increased cancer risk [140]. A Cochrane meta-analysis included a total of 50,623 participants, healthy or diagnosed with a specific disease, from 18 RCTs trials, which compared the effect of vitamin D supplementation/treatment (cholecalciferol, ergocalciferol, calcitriol, or alfacalcidiol, at any dose or regimen) vs. placebo on the risk of cancer. No conclusion could be drawn in terms of cancer prevention [141]. In a recently published, well-designed, multicenter, US-based RCT, 2259 subjects surgically treated for colorectal adenomas were randomized to receive daily vitamin D3 (1000 IU), calcium as carbonate (1200 mg), both or neither [142]. It was demonstrated that daily supplementation with vitamin D3, calcium, or both were ineffective in modifying the rate of recurrencies of colorectal adenomas over a period of 3–5 years [142].

As far as cancer mortality is concerned, there are conflicting results whether vitamin D supplementation reduces cancer-related mortality have been found [143, 144].

Although some evidence points to a possible role of treatment with active vitamin D analogs specifically in prostate cancer, the results of clinical studies are still underpowered and inconclusive, and require additional well-designed trials to establish efficacy [133, 145].

Secondary hyperparathyroidism is independently associated with poor prognosis in prostate cancer patients, especially when undergoing antiresorptive treatment for bone metastases [128, 146]. For this reason, in patients with metastatic bone disease, in whom treatment with agents such as bisphosphonates (i.e., zoledronic acid) or denosumab is commenced, vitamin D supplementation is recommended in order to normalize serum PTH levels and prevent side effects such as antiresorptive-induced hypocalcemia [146, 147].

In conclusion, although there is a high prevalence of low levels of vitamin D in cancer patients, insufficient evidence exists on the likely reduction of cancer incidence and mortality by vitamin D. The results of ongoing RCTs will possibly clarify these issues, in particular the optimal plasma concentrations of 25(OH)D to be achieved to get an effect for cancer prevention and/or treatment. In patients with prostate cancer undergoing antiresoptive therapy for the treatment of bone metastases, vitamin D supplementation should be undertaken to normalize serum PTH levels and decrease the risk of antiresorptive-related hypocalcemia. A similar recommendation may apply to patients with breast cancer commencing an antiresorptive therapy for prevention or treatment of bone metastases [148]. The efficacy and safety of active vitamin D analogs in certain types of cancer (i.e., prostate cancer) should be further explored.

## Vitamin D and chronic diseases in adults: considerations, recommendations and research agenda

In the vitamin D field, the strong mechanistic evidence for extra skeletal outcomes mainly ensues from in vitro studies, usually employing calcitriol in pharmacologic rather than physiologic doses, and from association studies, showing that low 25(OH)D levels (i.e., <50 nmol/l) are consistently associated with chronic diseases in prospective cohort studies with a dose–response relationship. Indirect evidence arises also from studies showing a direct trend between pathologic parameters or diseases, such as cardiovascular disorders, cancer, or autoimmune conditions, and the distance from the equator as well as fluctuations with seasonality (i.e., according to sub-optimal UV exposure).

Many cross-sectional studies have investigated the association between serum 25(OH)D levels and various parameters in health and disease. Although these studies have linked hypovitaminosis D to numerous disorders affecting different systems such as the cardiovascular, immune, endocrine/metabolic systems, they have not yet proven a causal relationship between a suboptimal vitamin D status and the onset and progression of these diseases. The Mendelian randomization method can be used in this setting, by using gene variants (i.e., polymorphisms) to make causal inferences in epidemiology and assess the causal effect of the exposure to different levels of serum 25(OH) on disease in non–experimental studies [149]. This method has been recently exploited in the vitamin D field to further assess the results of cross-sectional or longitudinal studies in large cohorts of subjects, where blood samples for genetic studies were available [149].

**Fig. 2:**
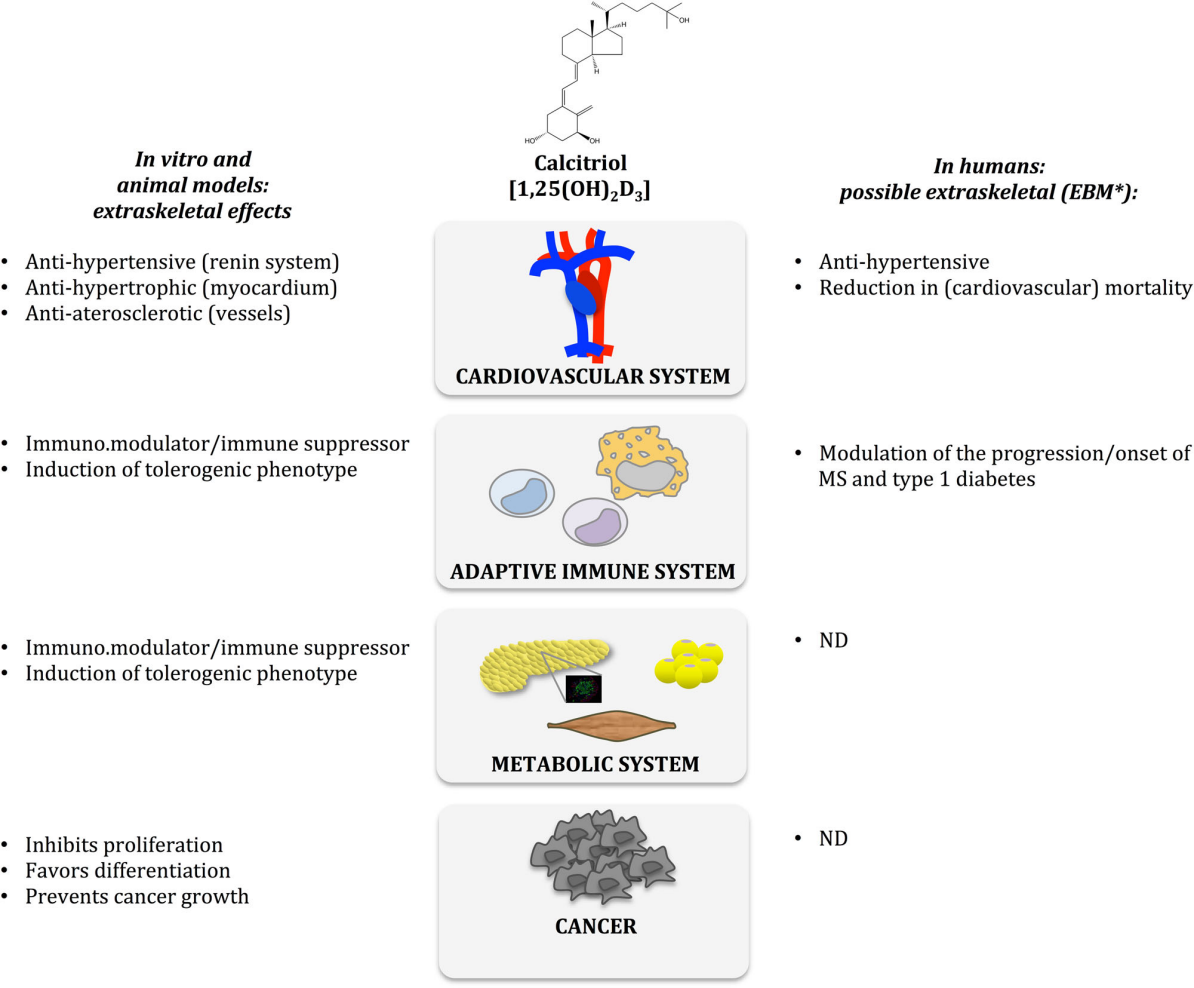
Calcitriol-mediated extraskeletal effects, as demonstrated in vitro and in vivo in animal models, likely mediating the possible extraskeletal effects in chronic diseases in humans (*Asterisk* shown according to Evidence Based Medicine’s levels 1b–2b; *ND* not demonstrated, i.e. level of evidence 2c and below). The extraskeletal effects have to be further confirmed given contradictory results in meta-analyses and randomized controlled trials (RCT)

These observations are still not supported by gross evidence in chronic diseases in humans, as demonstrated by the few available RCTs and the many meta-analyses and systematic reviews, often showing contradictory results (Fig. 2). Moreover, ecological evidence of the association between the prevalence of chronic diseases and UV irradiation arises from studies performed in the northern hemisphere, while these results are not reproduced in the southern hemisphere. There is definitely a preponderance of association studies over studies to demonstrate causality.

RCTs employing parental vitamin D compounds (cholecalciferol or calcidiol) in small cohorts of subjects have shown some results, yet far from leading to recommending for vitamin D use for primary or secondary prevention of extraskeletal diseases. Overall, the available RCTs analyzing the effect of vitamin D supplementation on specific extraskeletal outcomes is still scarce and clinical benefits from large RCTs of supplementation with vitamin D compounds, assessing both multiple outcomes and safety, have yet to be reported. The inclusion of participants with baseline serum 25(OH)D above the upper limit of deficiency (i.e., 50 nmol/l) could attenuate the effect of vitamin D on the main extraskeletal outcomes. Moreover, when employing large doses of vitamin D, safety has not usually been assessed. Monthly doses of vitamin D (or vitamin D supplements administered at even longer intervals) have been considered safe as far as classic side effects (i.e., hypercalciuria and hypercalcemia) are concerned and because of the long half-life of vitamin D. Nonetheless, this concept may have to be revised both because the half-life of vitamin D can be modulated by VDBP and because other active vitamin D-related metabolites can be produced during supplementation possibly modulating the main outcomes and/or mediating non-classic, adverse effects such as falls [7, 150–152]. These issues have not been taken into consideration in the trials assessing the effect of large doses of vitamin D in MS or in individuals at high risk of developing T1D.

Systematic reviews including large cohorts of patients belonging to cross-sectional or longitudinal studies or to meta-analyses of randomized intervention studies have shown an association with vitamin D status as measured by baseline or attained serum 25(OH)D levels and disease onset and progression in several contexts. One of the major concerns is represented by the U-curve relationship demonstrated by some of these studies, i.e., an increased risk both for low and high levels of serum 25(OH)D and disease or mortality. The lack of standardization of serum vitamin D assays [153], and the fact that meta analyses often combine trials including subjects with different starting baseline levels of serum 25(OH)D and employing different regimens of vitamin D to reach the same 25(OH)D target levels, are the major limitations of these studies. Indeed, the same attained level of serum 25(OH)D can be obtained after administering large doses at large intervals of time (bolus doses determining peak levels may be linked to unwanted effects, such as falls) or small doses, administered daily or weekly. Moreover, none of these studies take into account the possible production of active metabolites with short half-life, other than 25(OH)D or 1,25(OH)_2_D, which could contribute to the efficacy and safety profile of vitamin D supplementation [151].

For all the above reasons, it is still not possible to recommend the use or a dosage of vitamin D or related compounds as well as targets for serum 25(OH)D levels for the prevention or treatment or chronic, extra skeletal diseases, such as cardiovascular disorders, diabetes, autoimmune diseases, cancer and mortality. Nonetheless, supplementation with vitamin D along with antiresorptive therapy administered for the prevention of skeletal-related events or to treat bone metastases is needed, to control secondary hyperparathyroidism and prevent hypocalcemia.

Further studies are needed in this field (Table 2).

**Table 2:** Vitamin D and extraskeletal effects: research agenda

• To perform large randomized controlled trials simultaneously assessing multiple outcomes, assessing the efficacy of parental vitamin D compounds (cholecalciferol, ergocalciferol, calcidiol) or non-hypercalcemic active vitamin D analogs
• To employ and assess the efficacy of multiple regimens of parental vitamin D compounds
• To measure baseline and attained serum 25(OH)D levels by mass spectrometry, also in order to assess serum levels of other active and non-active intermediate/final vitamin D metabolites
• To enroll subjects with baseline serum 25(OH)D levels lower than 20 ng/ml (50 nmol/l)
• To assess safety in clinical trials evaluating non classical toxic effects (i.e. falls), besides classical toxic effects (hypercalciuria and hypercalcemia)
• To perform meta-analyses pulling together RCTs employing the same regimen, the same age group, and individuals with comparable baseline 25(OH)D levels
• To publish negative results of RCTs

It is advisable to perform large RCTs with multiple outcomes, assigning participants of similar age to different regimens of vitamin D supplementation, also comparing pro-hormones besides cholecalciferol, such as ergocalciferol (vitamin D2) and calcidiol [25(OH)D], which are supposed to give rise to different active intermediate metabolites with short half-life after supplementation that could be responsible for wanted or unwanted biologic effects. Levels of baseline and attained serum 25(OH)D should be measured with standard assays (i.e., mass spectrometry). Participants with low vitamin D levels (i.e., <50 nmol/l) should be enrolled. Safety (number of falls) should always be assessed as a secondary outcome. Studies employing multiple regimens of vitamin D, possibly against placebo, should be planned in order to test whether a dose–response relationship exists.

Furthermore, it is necessary to perform meta-analyses pulling together RCTs employing the same regimen, the same age group, and not just the target attained serum 25(OH)D, and individuals with comparable baseline 25(OH)D levels.

Negative trials should be published and included in meta-analyses.

While the administration of calcitriol should be avoided for the high risk of hypercalciuria and hypercalcemia, RCTs employing active, non-hypercalcemic vitamin D analogs should be carried out in patients with specific tumors, such as prostate cancer.

## Conclusion

The promising results from the growing literature on the associations between vitamin D and extraskeletal chronic is not matched by the results obtained in intervention studies. To prove a causal relationship and recommend the use of vitamin D-related compounds in extra skeletal diseases, more trials are needed to demonstrate that maintaining 25(OH)D levels within a certain range may be useful and safe in both the prevention and treatment of these diseases.
